# How Far is between Cancer and Health?

**Published:** 2019-05

**Authors:** Chunsong HU, Tengiz TKEBUCHAVA, Qinghua WU, Dayi HU

**Affiliations:** 1. Department of Cardiovascular Medicine, Nanchang University, Nanchang 330006, China; 2. Boston TransTec, LLC, Boston, MA 02459, USA; 3. Cardiovascular Center, Peking University People’s Hospital, Beijing 100044, China

## Dear Editor-in-Chief

With the rapid economic and social development, the large epidemic of major non-communicable diseases (mNCDs) due to unhealthy lifestyles lead to a heavy burden and a tremendous challenge in both advanced and developing countries. Just as “the three big mountains”, cardiovascular disease, diabetes, and cancers affect greatly healthy life expectancy. Health is everyone’s goal, it’s also a dream from reality to the future (1). In fact, there are only 4.3% population with entire health. According to updated data from 347 national cancer registries collected by the Chinese National Cancer Center (NCC), cancer incidence of each person in Chinese urban residents is more than 35% throughout their lives, and about ten thousand people are diagnosed with cancer every day. Therefore, how to keep one’s health and how far between cancer and health?

### 1. Health: general formula and R factor

Traditionally, the cause of cancer is thought to be closely related to genetic factors and lifestyle including the environment and is mainly caused by unhealthy lifestyle. People found that the majority of cancers is due to “bad luck”, that is, random mutations arising during DNA replication. Recently, 66% of cancer is caused by the errors or mutations occur during DNA replication (2), referred to as “replication factor” (R factor). However, we think that the etiology and occurrence of mNCDs, especially cancer, and related CDC strips (3), are associated primarily with unhealthy lifestyle.

We agreed that, despite healthy lifestyle, “bad luck” or R factor still has a chance in the occurrence of cancer by a certain percentage. However, this does not negate the preventive effect of healthy lifestyle on cancer. For individuals, if they are leading unhealthy lifestyle, the incidence of cancer and other mNCDs will be significantly increased, the risk of early death will also increase. However, with an established healthy lifestyle, most cancers can be prevented, at least their risks will be controlled. It’s easy to realize lifespan extension and healthy aging by dietary restriction and supplement of key nutritional elements.

**Fig. 1:**
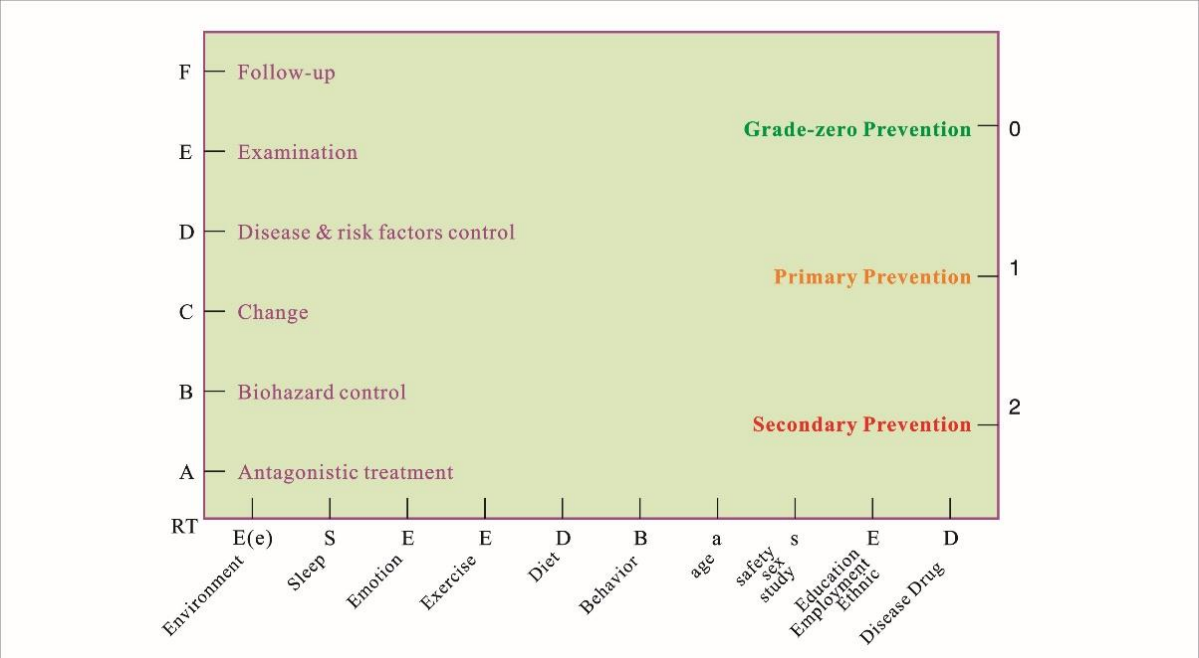
General formula (GF) for non-communicable disease (NCD) (equation of health and longevity) This figure shows tips of GF, whoever follows and practices it, who will realize health and longevity

The general formula or health and longevity equation have been set up for management and prevention of human disease, that is, health & longevity = RT-ABCDEF + E(e)SEED-BasED + 210 (Fig. 1) (4, 5). It should be added a new interpretation due to new studies on R factor. We need to prevent DNA replication errors or mutations, i.e. R factor-induced cancer. Although it is random, with the so-called “bad luck”, the probability of cancer will be greatly reduced by the general formula, for example, controlling internal or external environment by decreasing air pollution. Thus, it is very necessary to promote positively SEEDi^1.0–3.0^ strategies in the global (5), help to decrease greatly the occurrence of cancer and other mNCDs.

### 2. Cancer Risk: Lifestyle versus R Factor

**Fig. 2:**
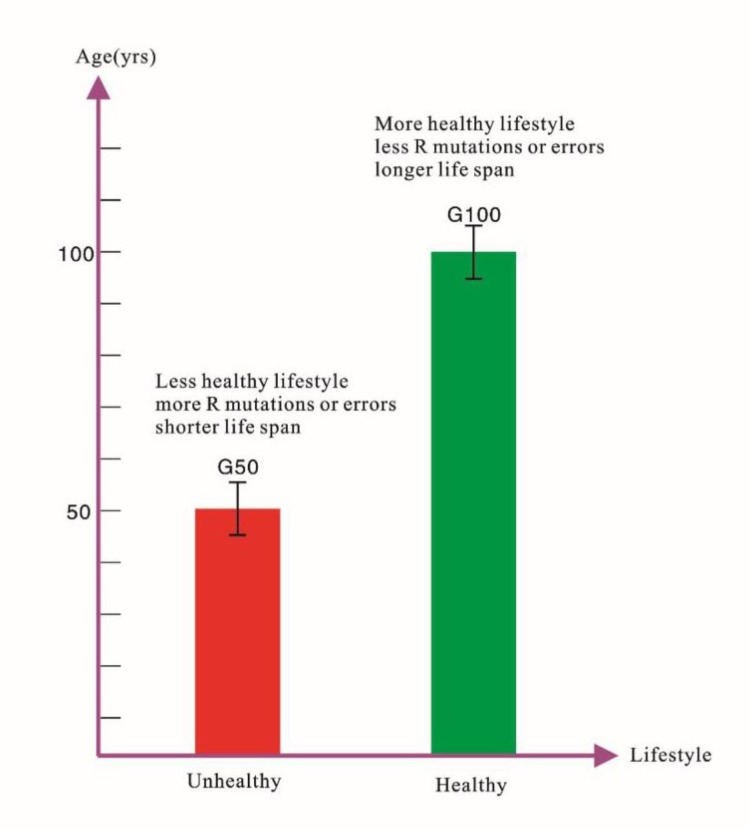
Cancer risk and life span: two groups (G50 and G100) data on lifestyle versus R factor

What’s the exact cancer risk? Generalized internal environment which includes patients’ family history or genetically diseased organs, as well as inflammation or lesions, is highly associated with cancer risk. Although there is R factor (2), we still think that the etiology and occurrence of cancer and related CDC strips are associated primarily with unhealthy lifestyle, which includes external and generalized internal environment. This can be explained and supported by two groups of data (Fig. 2).

A group of longevity (average 100 yr old, G100) and a group of young deaths (average 50 yr old, G50). Most of the causes of death in G100 are non-cancer, which include disease-free death or death from cardiovascular disease, lung infection or other accidents. The proportion of deaths from cancer is lower than in G50. Their longevity is due to simple and healthy lifestyle (2), some have family histories; due to their longer life spans, theoretically, the chance of R factor-induced cancer is greater. However, the G100 is suffering less from cancer; G50 died of a higher proportion of cancer, with the exception of familial history of cancer, mostly due to unhealthy lifestyle (3), such as staying up late and heavy smoking. Because they are younger, theoretically the risk of R factor-induced cancer should be less, but in fact, the proportion of cancer was significantly higher among these individuals than G100. Cancer is mainly related to lifestyle even if there is also “bad luck” or R factor (2).

All in all, when somebody asks you how far between cancer and health, the humor answer is 1m (mutation) or Nm (not mutation), which lays on one’s lifestyle. In fact, most of subjects are under middle-status between cancer and health. Since lifestyle has already become a key target for preventing and controlling cancer, it’s very necessary to conduct positively Grade-zero prevention (1) and SEEDi^1.0–3.0^ strategies for keeping good health (5). As a tool of precise medicine, people may expect decreasing DNA replication errors or mutations by methods of biological structure-editing (6), therefore, as to control R factor effectively and decrease cancer risk in the future.
